# Excessive Endoplasmic Reticulum Stress Correlates with Impaired Mitochondrial Dynamics, Mitophagy and Apoptosis, in Liver and Adipose Tissue, but Not in Muscles in EMS Horses

**DOI:** 10.3390/ijms19010165

**Published:** 2018-01-06

**Authors:** Krzysztof Marycz, Katarzyna Kornicka, Jolanta Szlapka-Kosarzewska, Christine Weiss

**Affiliations:** 1 Department of Experimental Biology, Wroclaw University of Environmental and Life Sciences, 50-375 Wroclaw, Poland; kornicka.katarzyna@gmail.com (K.K.); jolanta.szlapka@gmail.com (J.S.-K.); 2 Wroclaw Research Centre EIT+, ul. Stabłowicka 147, 54-066 Wrocław, Poland; 3 PferdePraxis Dr. Med. Vet. Daniel Weiss, Postmatte 14, CH-8807 Freienbach, Switzerland; d.weiss@horsedoc.ch

**Keywords:** metabolic syndrome, apoptosis, insulin resistance, mitochondria, autophagy

## Abstract

Nowadays, endocrine disorders have become more frequent in both human and veterinary medicine. In horses, reduced physical activity combined with carbohydrate and sugar overload may result in the development of the so-called equine metabolic syndrome (EMS). EMS is characterized by insulin resistance, hyperinsulinemia, elevated blood triglyceride concentrations and usually obesity. Although the phenotypic features of EMS individuals are well known, the molecular mechanism underlying disease development remains elusive. Therefore, in the present study, we analyzed insulin-sensitive tissues, i.e., muscles, liver and adipose tissue in order to evaluate insulin resistance and apoptosis. Furthermore, we assessed mitochondrial dynamics and mitophagy in those tissues, because mitochondrial dysfunction is linked to the development of metabolic syndrome. We established the expression of genes related to insulin resistance, endoplasmic reticulum (ER) stress and mitochondria clearance by mitophagy using RT-PCR and Western blot. Cell ultrastructure was visualized using electron transmission microscopy. The results indicated that adipose tissue and liver of EMS horses were characterized by increased mitochondrial damage and mitophagy followed by triggering of apoptosis as mitophagy fails to restore cellular homeostasis. However, in muscles, apoptosis was reduced, suggesting the existence of a protective mechanism allowing that tissue to maintain homeostasis.

## 1. Introduction

Equine metabolic syndrome (EMS) is an increasingly common endocrine disease that is a constellation of clinical abnormalities associated primarily with insulin resistance. It is caused by reduced physical activity combined with carbohydrate and sugar overload, leading as consequence to insulin resistance (IR) and obesity; however, recent findings suggest that obesity and/or regional adiposity per se should not be used as diagnostic criteria [1]. The most characteristic features of the EMS horse phenotype are fasting hyperinsulinemia, elevated blood triglyceride concentrations and inadequate insulin response to external glucose administration (oral or intravenous). 

Severe obesity and/or regional adiposity, in addition to being a characteristic symptom in EMS diagnosis, is an important metabolic factor affecting the physiological and cytophysiological condition of peripheral tissue, inducing, among others, inflammation, autophagy and apoptosis. It has been established that adipose tissue of both horses and humans affected with EMS secretes pro-inflammatory cytokines and adipokines to the peripheral blood, which might be a major factor contributing to insulin resistance development [2,3,4].

We demonstrated in our previous research [5] that in insulin-resistant EMS ponies, “cresty neck”-derived adipose tissue secreted abundantly pro-inflammatory cytokines, adipokines and hormones. This in turn leads to a low-grade systemic inflammation, which is crucial in the context of laminitis development. Other authors also found higher protein levels of tumor necrosis factor α (TNF-α), interleukin 1β (IL-1β) and IL-6 in the vicinity of the nuchal ligament in EMS horses; however, no statistical correlation was observed at the mRNA level in hyperinsulinemic horses compared to healthy individuals of normal weight. Moreover, no significant differences were found between the expression of TNF-α, IL-1β, IL-6 and MCP-1 in different fat depots of EMS horses versus healthy individuals [6]. This is surprising given the fact that EMS is generally identified with adipose tissue inflammation. However, increased fat content in liver and muscles is suspected to be a major factor leading to the development of systemic proinflammatory state, due to the elevated expression of TNF-α combined with suppressor of cytokine signaling 3 (SOCS3) and Toll-like receptor 4 (TLR4). In human metabolic syndrome, abnormal fat accumulation, especially in the liver, is believed to be mainly responsible for inducing local inflammation, due to its lipotoxicity, which in turn causes liver fibrosis and initiates hepatocyte apoptosis. The progressive inflammation and oxidative stress of hepatocytes, adipocytes and myocytes, together with progressive apoptosis in the course of EMS may in turn be the factors that induce autophagy or/and mitophagy to rescue stressed cells.

Oxidative stress (OS) has been implicated in the progression of metabolic syndrome, diabetes and their complications in humans [7,8]. Excessive accumulation of reactive oxygen species (ROS) has been shown to disrupt the insulin signaling pathway, thereby contributing to insulin resistance (IR). It has been shown that dyslipidemia, hyperglycemia endoplasmic reticulum (ER) stress and nitric oxide synthase are responsible for ROS overproduction. Moreover, mitochondrial dysfunction is usually associated with increased ROS secretion by organelles or indirectly by sending signals to the ER surface, which in turn, produces even more ROS. Furthermore, it can trigger systemic inflammation characteristic of metabolic syndrome and diabetes. However, there are only limited data regarding the degree of OS in EMS horses. In the present study, we have determined serum levels of ROS, nitric oxide and superoxide dismutase to assess whether OS is characteristic of EMS.

Autophagy is recognized as a mechanism that is responsible for the degradation of impaired cell organelles and their utilization and plays a crucial role in maintaining cellular cytophysiological functions [9]. On the other hand, the term mitophagy relates to the removal of dysfunctional, damaged mitochondria to maintain energy balance and homeostasis in cells. Activation of an autophagic or lysosomal system can eliminate oxidized cell components in the OS response. Recent data suggest that nutrient-induced stress of the ER, which is an important factor in developing obesity-associated inflammation, may be a potent inducer of autophagy, enabling recycling damaged organelles, including mitochondria [10,11]. In our previous study, we have demonstrated that macroautophagy and mitophagy are involved in the survival of EMS-derived progenitor cells. Therefore, autophagy, as an important quality control of organelles, may be a key mechanism that allows protecting myocytes, hepatocytes and adipocytes in the metabolic syndrome. However, it has been demonstrated in obese type II diabetes patients that the removal of defective mitochondria via mitophagy may be a crucial mechanism that rescues mitochondria from metabolic overload, which results in incomplete β-oxidation, accumulation of toxic lipid intermediates and oxidative stress [12]. However, recent research in humans has indicated that in the state of obesity, the autophagy process is indeed upregulated in adipose tissue, but can be severely impaired. In our previous research, we have demonstrated the importance of autophagy in adipose stem cells isolated from EMS horses as a protective mechanism, allowing those cells to maintain multipotency. In this study, we aimed to investigate autophagy in insulin-sensitive tissues from EMS horses and for the first time examine insulin resistance at molecular levels in those animals. To our knowledge, this is the first research describing the molecular consequences of EMS in the liver, muscle and adipose tissue. Our main goal was to examine how EMS affects those tissues’ metabolism in comparison to healthy individuals as many data exist about that phenomenon in humans. In the present research, we unraveled for the first time EMS-related deterioration of insulin-sensitive tissues. We evaluated muscles, adipose tissue, liver, as well as the cross-talk between apoptosis, inflammation, ER stress and autophagy in EMS horses to revise these hypotheses for the first time. We determined cyclin-dependent kinase inhibitor 1 (p21), tumor protein p53 (p53), alpha serine/threonine-protein kinase (BAX)/B-cell lymphoma 2 (Bcl-2) ratio, p62, Beclin-1, PINK PARKIN and microtubule-associated protein 1A/1B-light chain 3 (LC-3) levels, as well as the presence of autophagosomes characteristic of both autophagy and autophagy-induced apoptosis. In addition, we assessed markers of mitochondrial dynamics and endoplasmic reticulum stress. Our results showed a positive correlation between apoptosis, ER stress and impaired mitophagy in the liver and adipose tissue, but not in muscles.

## 2. Results 

### 2.1. Oxidative Stress and Inflammation 

**Figure 1:**
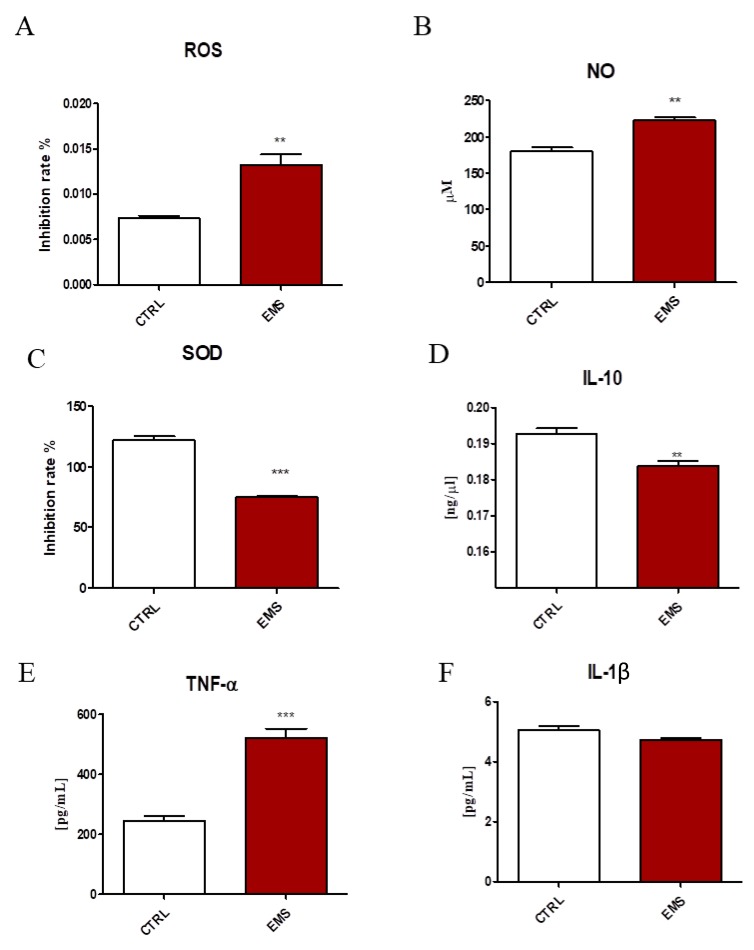
Oxidative stress and cytokines levels in the serum of healthy (CTRL) and EMS horses. Using commercially available, spectrophotometric assays, ROS (**A**), NO (**B**) and SOD (**C**) activity was assessed. The cytokine profile was evaluated with ELISA for IL-10 (**D**), TNF-α (**E**) and IL-1β (**F**). Results expressed as the mean ± standard deviation (S.D.) ** *p* < 0.01, *** *p* < 0.001.

Oxidative stress factors and cytokine levels were established in serum of healthy and EMS individuals. Both ROS (Figure 1A) and NO (Figure 1B) levels were markedly increased in the serum of EMS horses (*p* < 0.01). On the other hand, the activity of antioxidative enzyme superoxide dismutase (SOD) was significantly downregulated (Figure 1C, *p* < 0.001). Using ELISA, the amount of interleukin 10 (IL-10), TNF-α and IL-1β in serum was established. The amount of IL-10 (Figure 1D, *p* < 0.01) was diminished, while TNF-α (Figure 1E, *p* < 0.001) increased in EMS horses. No statistically-significant differences between groups were observed in the levels of IL-1β (Figure 1F). 

### 2.2. Insulin Resistance 

**Figure 2:**
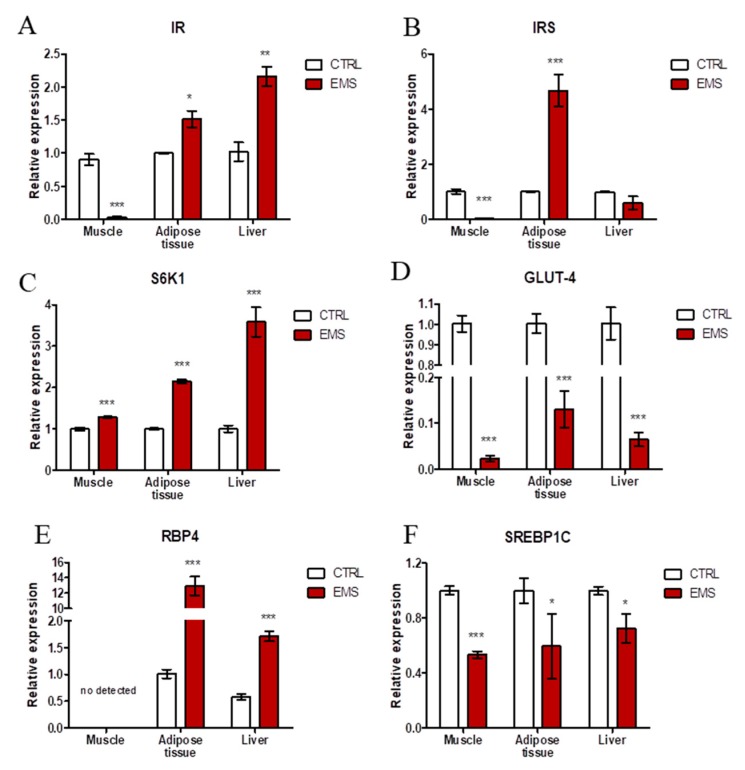
Expression of insulin resistance-related genes in muscle, adipose tissue and liver of horses. Using RT-PCR, the expression of IR (**A**), IRS (**B**), S6K1 (**C**), GLUT-4 (**D**), RBP4 (**E**) and SREBP1C (**F**) was investigated. Results expressed as the mean ± S.D. * *p* < 0.05, ** *p* < 0.01, *** *p* < 0.001.

Using RT-PCR, expression of insulin-resistance-related genes was evaluated in muscles, liver and adipose tissue of healthy and EMS horses. Insulin receptor (IR) expression was barely detectable in muscles of EMS horses, while in adipose and liver, it was increased (Figure 2A, *p* < 0.05 and *p* < 0.001 respectively). Similarly, insulin receptor substrate expression (Figure 2B) was markedly reduced in muscles of EMS horses (*p* < 0.001), while in adipose tissue, the mRNA level of insulin receptor substrate (IRS) was markedly increased (*p* < 0.001). No significant differences were noted in IRS amount in liver between investigated groups. Expression of ribosomal protein S6 kinase beta-1 (S6K1) (Figure 2C) was augmented in all tissues isolated from EMS individuals (*p* < 0.001). On the contrary, levels of glucose transporter 4 (GLUT-4) were significantly decreased (*p* < 0.001) in all tissues from EMS horses as shown in Figure 2D. Retinol binding protein 4 (RBP4) (Figure 2E) was not detected in muscles; however, it was upregulated in adipose tissue and liver of EMS horses (*p* < 0.001). RT-PCR for sterol regulatory element binding protein 1c (SREBP1C) (Figure 2F) revealed its decreased expression in the muscles (*p* < 0.001), adipose tissue (*p* < 0.05) and liver (*p* < 0.05) of EMS animals. 

### 2.3. Apoptosis

**Figure 3:**
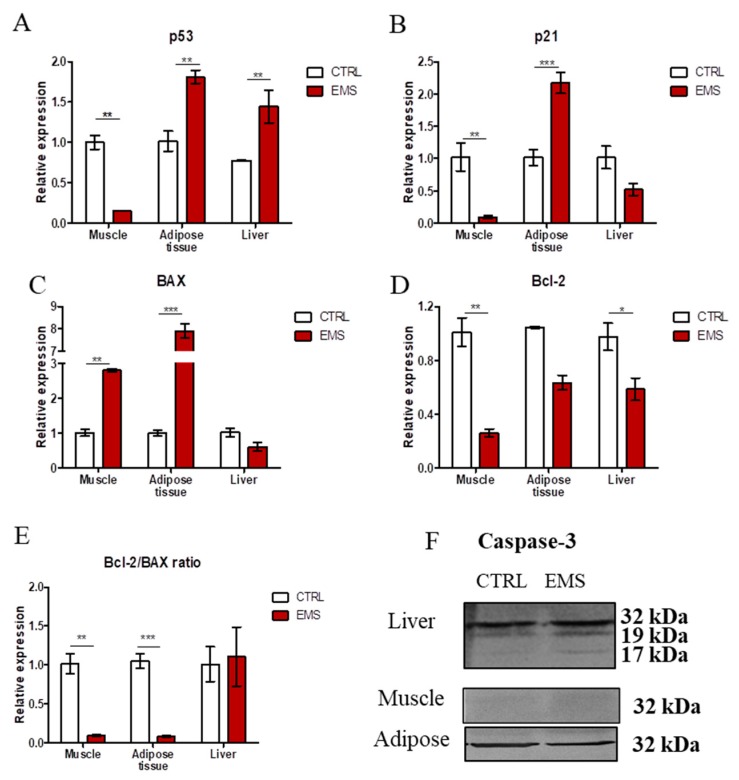
Evaluation of apoptosis in muscle, adipose tissue and liver of healthy (CTRL) and EMS individuals. Using RT-PCR, we evaluated the expression of p53 (**A**), p21 (**B**), BAX (**C**), Bcl-2 (**D**) and the Bcl-2/BAX ratio (**E**). Moreover, the amount of caspase-3 in samples was established by Western blot analysis (**F**). Results expressed as the mean ± S.D. * *p* < 0.05, ** *p* < 0.01, *** *p* < 0.001.

All tissues were homogenized in Tri Reagent and submitted for RNA isolation. Using RT-PCR, expression of apoptosis-related genes was studied in investigated specimens. Expression of p53 (Figure 3A) in muscles was significantly reduced in EMS horses (*p* < 0.01). On the contrary, p53 mRNA was upregulated in both adipose tissue (*p* < 0.01) and liver (*p* < 0.01) in those individuals. Expression of p21 (Figure 3B) was significantly diminished in the muscle of EMS horses in comparison to healthy subjects (*p* < 0.01). On the contrary, in the case of adipose tissue, p21 transcript levels were markedly increased (*p* < 0.001). There was no statistical significance in the expression of p21 in liver of healthy and EMS horses. BAX (Figure 3C) expression was significantly upregulated in muscle (*p* < 0.01) and adipose tissue (*p* < 0.001) of EMS horses, while no significant differences were noted in its expression in the liver between groups. Expression of anti-apoptotic Bcl-2 (Figure 3D) was significantly diminished in muscle (*p* < 0.01) and adipose tissue (*p* < 0.001) of EMS horses. The ratio of Bcl-2/BAX was significantly decreased in muscle and liver of EMS horses (Figure 3E). Furthermore, using Western blot, the amount of caspase 3 in samples was investigated. Results are shown in Figure 3F. Interestingly, only in the liver, cleavage of caspase 3 was observed. 

### 2.4. Endoplasmic Reticulum (ER) Stress

**Figure 4:**
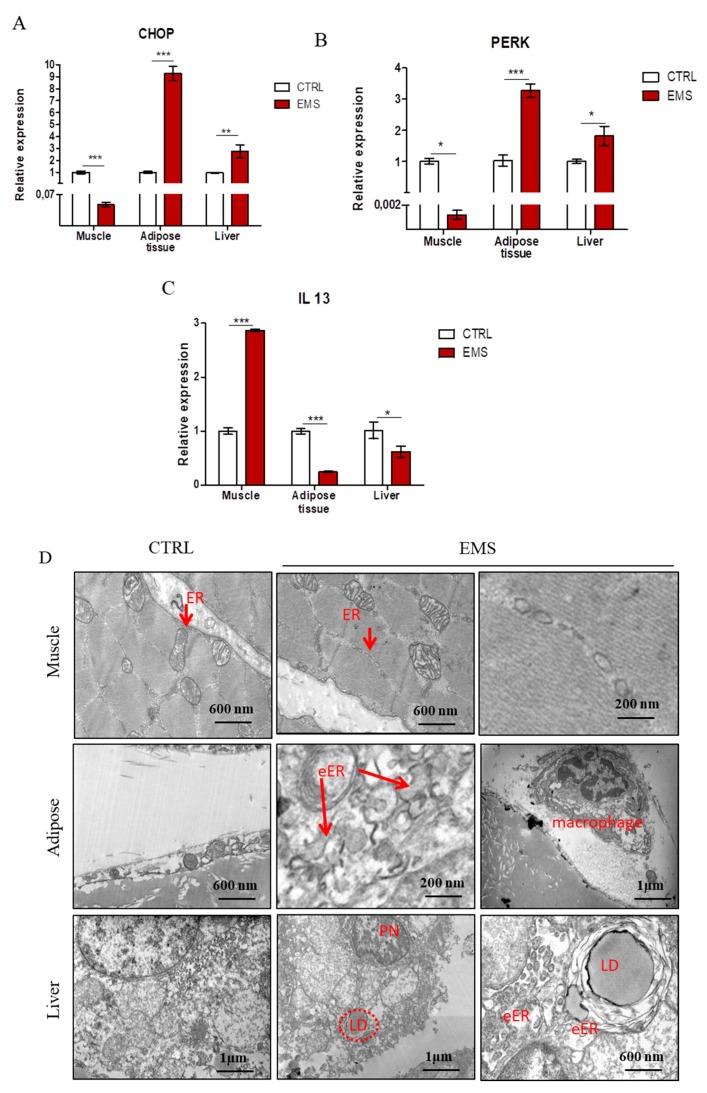
Investigation of ER stress in tissues from healthy and EMS horses. Using RT-PCR, the expression of CHOP (**A**), PERK (**B**) and IL-13 (**C**) was examined. Furthermore, TEM images allowed for visualization of cellular ultrastructure and detection of organelles’ impairment (**D**). Abbreviations: ER, endoplasmic reticulum; eER, enlarged endoplasmic reticulum; LD, lipid droplets; PN, pyknotic nucleus. Results expressed as the mean ± S.D. * *p* < 0.05, ** *p* < 0.01, *** *p* < 0.001.

In order to evaluate ER stress in investigated tissues, we performed RT-PCR for CCAAT-enhancer-binding protein homologous protein (CHOP) (Figure 4A), eukaryotic translation initiation factor 2-alpha kinase 3 (PERK) (Figure 4B) and interleukin 13 (IL-13) (Figure 4C). The obtained results indicated an increased expression of CHOP in adipose tissue (*p* < 0.001) and liver (*p* < 0.01) while decreased in muscles (*p* < 0.001) of EMS horses. The amount of PERK mRNA was downregulated in muscles of EMS horses (*p* < 0.05). However, it was upregulated in those individuals’ adipose tissue (*p* < 0.001) and liver (*p* < 0.05). Expression of IL-13 (Figure 4D) was significantly upregulated in muscles (*p* < 0.001) and downregulated in adipose tissue (*p* < 0.001) and liver (*p* < 0.05) of EMS individuals. To further investigate ER condition, we performed transmission electron microscope (TEM) analysis of tissue samples (Figure 4D). In TEM images, no marked differences were noted in ER ultrastructure between groups. In the case of adipose tissue, in the control group, ER was barely visible; however, in the EMS group the volume of ER structures was increased, which is a typical consequence of unfolded protein stress (indicated as eER (enlarged ER)). Moreover, macrophages infiltration was noted in adipose tissue of EMS animals. A similar phenomenon was observed in livers of EMS horses, as enlarged ER was noted as well. Moreover, they were characterized by enhanced accumulation of lipid droplets. Furthermore, pyknotic nuclei were noted, which indicates increased hepatocytes apoptosis.

### 2.5. Autophagy

**Figure 5:**
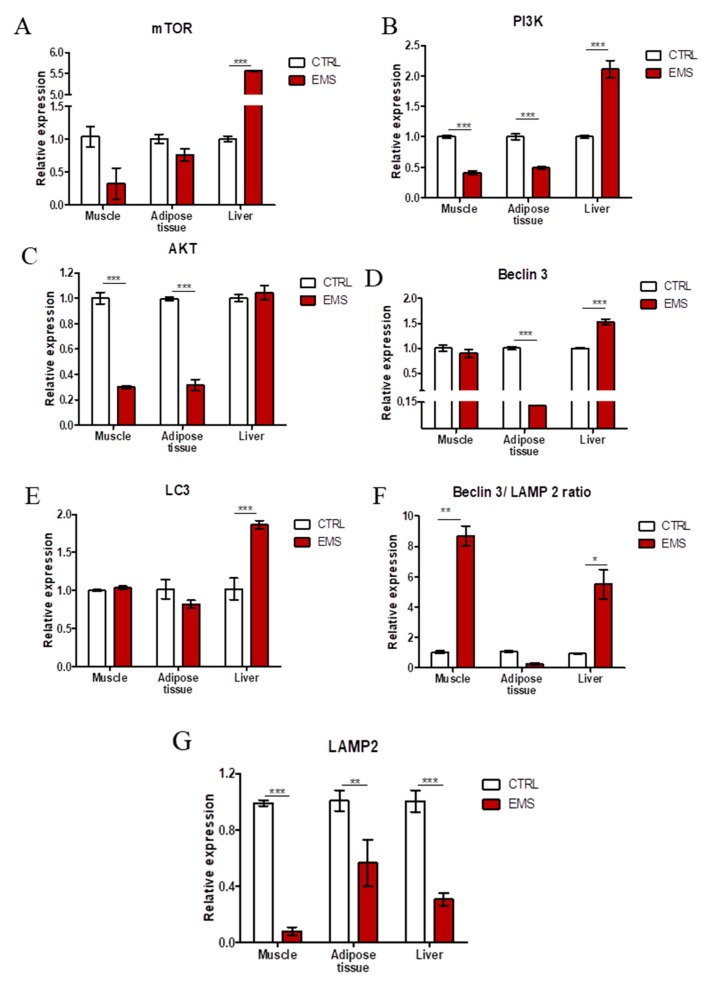

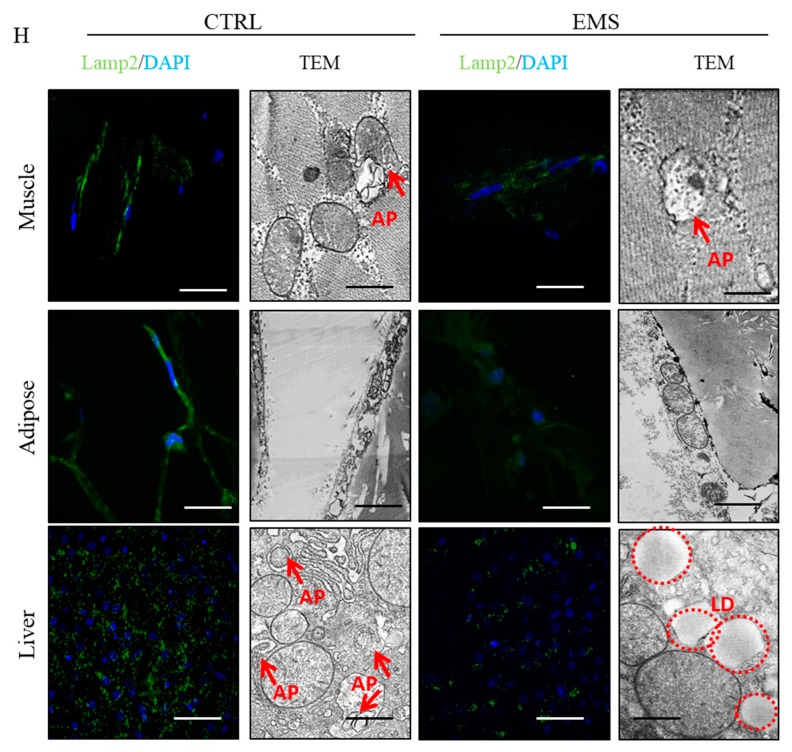
Evaluation of autophagy in muscle, adipose tissue and liver from healthy and EMS individuals. Using RT-PCR, the expression of mTOR (**A**), PI3K (**B**), AKT (**C**), Beclin-3 (**D**), LC3 (**E**), the ratio of Beclin/LAMP2 (**F**) and LAMP2 (**G**) was investigated. Using immunofluorescence, intracellular localization of LAMP2 in samples was examined (**H**). TEM analysis allowed for visualization of autophagosomes’ formation (indicated with red arrows (**I**)). LD, lipid droplets; AP, autophagosomes. Results expressed as the mean ± S.D. * *p* < 0.05, ** *p* < 0.01, *** *p* < 0.001. Scale bars: confocal 20 µm, transmission electron microscope (TEM) 200 nm.

Autophagy-related genes’ expression was evaluated using RT-PCR. No differences between groups were noted in the mechanistic target of rapamycin (mTOR) (Figure 5A) expression in muscle and adipose tissue samples. However, in liver, it was upregulated in the EMS group (*p* < 0.001). Phosphatidylinositol-4,5-bisphosphate 3-kinase (PI3K) (Figure 5B) mRNA levels were downregulated in muscles (*p* < 0.001) and adipose tissue (*p* < 0.001) of EMS horses. On the contrary, in liver, PI3K (Figure 5C) expression was markedly upregulated (*p* < 0.001) in comparison to the control group. Similarly, AKT expression was downregulated in muscle (*p* < 0.001) and adipose tissue (*p* < 0.001) of EMS animals. No differences in AKT mRNA amount were noted in the liver. In turn, no differences were noted in the expression of Beclin-3 (Figure 5D) in muscle samples. In adipose tissue, Beclin-3 expression was diminished (*p* < 0.001), while in liver enhanced (*p* < 0.001) in the EMS group. LC-3 (Figure 5E) expression levels were comparable in muscle and adipose tissue, although it was significantly upregulated in liver of EMS animals (*p* < 0.001). The ratio of Beclin-3/LAMP2 (Figure 5F) expression was greatly increased in muscle and liver of EMS individuals (*p* < 0.01 and *p* < 0.05). No differences were noted in the ratio calculated for adipose tissue samples. Lysosome-associated membrane protein 2 (LAMP2) (Figure 5G) expression was greatly diminished in all tissues from EMS horses. Using fluorescence staining for LAMP2 and confocal microscope imaging, we evaluated the amount of LAMP2 in tissues. Immunofluorescence staining results confirmed PCR data, as a low intensity of the LAMP2 signal was noted in tissues from EMS horses. Moreover, using TEM, we visualized autophagosomes’ formation in samples. Autophagosomes are indicated with red arrows in Figure 5H.

### 2.6. Mitophagy and Mitochondrial Dynamics

**Figure 6:**
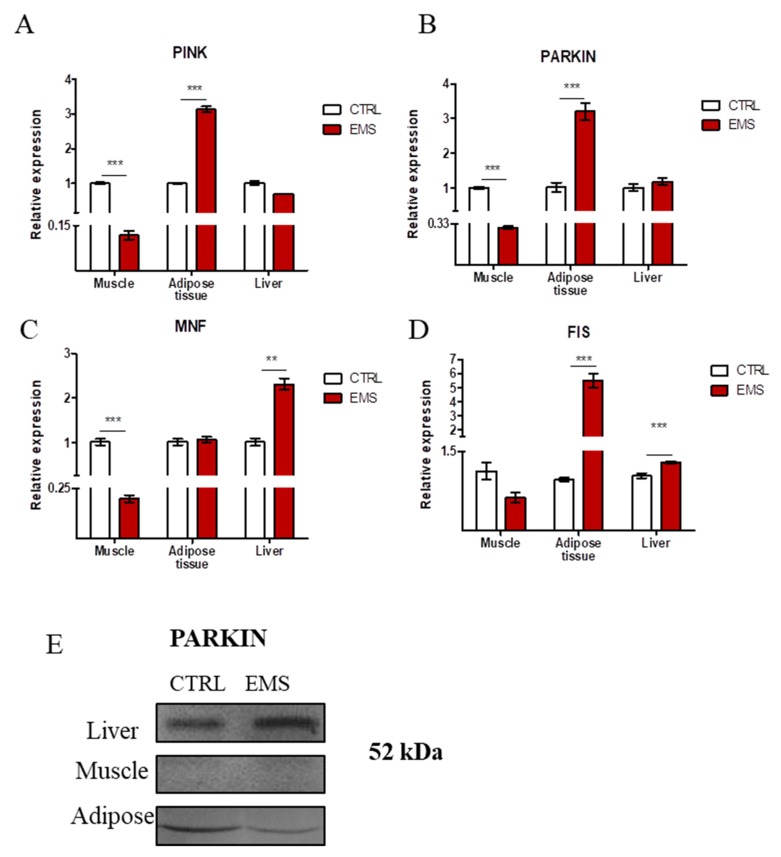

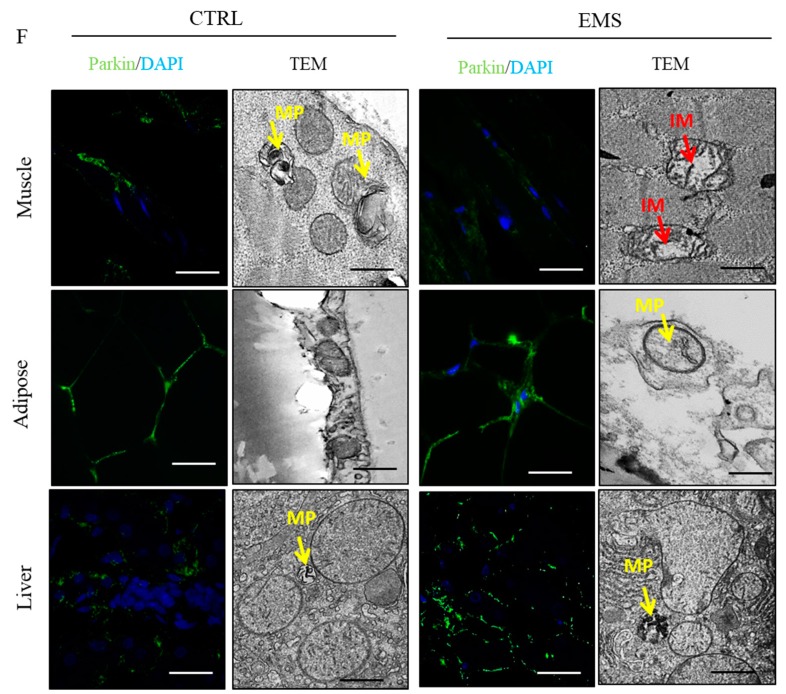
Evaluation of mitophagy and mitochondrial dynamics in tissues from healthy and EMS-diagnosed horses. Using RT-PCR, the expression of PINK (**A**), PARKIN (**B**), MNF (**C**) and FIS (**D**) was investigated. PARKIN amount was also examined by Western blot (**E**) and immunofluorescence staining (**F**). TEM imaging (**F**) allowed for visualization of mitophagosomes’ formation (indicated with yellow arrows) and mitochondria morphology impairment (indicated with red arrows). MP, mitophagosomes; IM, impaired mitochondria. Results expressed as the mean ± S.D. ** *p* < 0.01, *** *p* < 0.001. Scale bars: confocal 20 µm, TEM 100 nm.

Using RT-PCR, we investigated the expression of genes involved in the progression of mitophagy: PINK (Figure 6A) and Parkin ligase (PARKIN) (Figure 6B). No differences were noted in PINK expression in liver; however, it showed decreased expression in muscle of EMS horses (*p* < 0.001). Interestingly, in the EMS group, PINK expression was upregulated in adipose tissue (*p* < 0.001). The same trend was observed in PARKIN expression: it was decreased in muscle (*p* < 0.001) and increased in adipose tissue (*p* < 0.001) of EMS horses. No differences were noted in PARKIN expression in liver samples. To evaluate mitochondrial dynamics in tissues, we investigated the expression of genes related to mitochondrial fusion and (Figure 6C) fission (Figure 6D). Expression of mitofusin 1 (MNF) was downregulated in muscles (*p* < 0.001), while increased in liver (*p* < 0.01) of EMS horses. No differences in MNF mRNA amount were noted in adipose tissue samples between groups. mitochondrial fission 1 protein (FIS) expression displayed no differences in muscles of horses, while it was upregulated in both adipose tissue (*p* < 0.001) and liver (*p* < 0.001) of those individuals. Furthermore, PARKIN amount was evaluated using Western blot as shown in Figure 6E. Moreover, using immunofluorescence staining for PARKIN, its cellular localization was visualized in tissues (Figure 6F). The obtained results supported PCR data as the strongest signal intensity was observed in adipose tissue and liver of EMS horses. Furthermore, using TEM, we visualized the formation of mitophagosomes (indicated with yellow arrows, Figure 6F). The obtained data indicated mitochondria deterioration (disarrayed cristae, swollen shape) in muscles of EMS horses (indicated with red arrows in Figure 6F). 

## 3. Discussion

The major pathological sign of equine metabolic syndrome is insulin resistance of muscles, adipose tissue and liver, as a result of diet overloaded with carbohydrates and reduced physical activity [13]. All these factors impair insulin signaling, which is a major component of endocrine disorders, including EMS.

We measured ROS, SOD and NO levels in horse serum to further investigate the relationships between OS and EMS. Many research works have indicated that diabetic patients show increased ROS production in circulation and reduced antioxidant protection [14,15]. It has been demonstrated that oxidative stress enhances insulin resistance in rat models [16]. This is consistent with our data, as we have observed increased serum levels of ROS and NO in EMS individuals. It was shown that NO synthase (NOS) was increased in the arteries of diabetic rats [17]. Furthermore, ROS has been proven to be especially harmful to pancreatic beta cells, as they have a low content of antioxidants, such as SOD or catalase [18]. In the present study, we observed decreased levels of serum SOD, which indicated reduced antioxidative protection in EMS horses. This is consistent with the results of Isogawa et al. [19], who observed that serum SOD activity negatively correlated with the body mass index (BMI) of metabolic syndrome subjects. In this setting, IR may serve as a compensatory mechanism, protecting cells from glucose and fatty acid uptake, and thus oxidative damage [20,21]. Recent studies have indicated that physical activity and dietary restriction may ameliorate OS [22]. It was demonstrated that antioxidant intake is correlated with increased risk of diabetes [23]. Hence, supplementation of horse diet with vitamins and free radical scavengers, combined with physical activity, may decrease circulation levels of ROS and, as a consequence, reduce inflammation. 

The impairment of insulin signaling is not without significance for endoplasmic reticulum (ER) stress, as well as mitochondrial biogenesis and dynamics [24,25], which are strongly associated with EMS. Here, we have observed a reduced expression of GLUT4 and sterol regulatory element-binding transcription factor 1 (SREBF1) in muscles, adipose tissue and liver in EMS horses and a simultaneous upregulated mRNA transcription of ribosomal protein S6 kinase 1 (S6K1). Our results indicate impaired insulin sensitivity, dysfunction of fatty acid homeostasis, as well as adiposity of muscles, adipose tissue and liver in EMS horses compared to healthy donors. Furthermore, we have found increased levels of S6K1 transcripts in all investigated tissues in EMS horses. Higher levels of S6K1 mRNA were reported as a key factor determining insulin resistance development in conditions of nutrient overload [26]. Interestingly, the increased expression of retinol-binding protein (RBP) 4, which positively correlates with age, obesity and insulin resistance, has been recorded in adipose tissue and liver, but not in muscles in EMS horses; nevertheless, such expression has been previously reported in rodent muscles [27]. Furthermore, it was shown that increased expression of RBP4 led to apoptosis via STRA6 signaling [28]. Here, we have observed a positive correlation between the increased levels of RBP4 transcripts in adipose tissue and liver, which indicated progressive apoptosis. The upregulation of both RBP4 and p53 in adipose tissue and liver may be correlated with insulin resistance, since the inhibition of p53 transcripts was reported to improve insulin sensitivity and decrease the expression of proinflammatory cytokines in mice, however, with type 2 diabetes-like disease [29]. The progressive p53-related apoptosis in adipose tissue and liver of EMS horses may be caused by high endoplasmic reticulum (ER) stress, which leads to disruption of ER homeostasis and eventually β-cell death [30]. Recently, it has been suggested that chronic hyperglycemia and hyperlipidemia are major components responsible for ER stress and are both characteristic of EMS condition. Here, we have observed significantly higher levels of CHOP (C/EBP homologous protein) and PERK (eukaryotic translation initiation factor 2-alpha kinase 3) transcripts in both adipose tissue and liver, but not in muscles of EMS horses. This positively correlated with upregulated p53 expression in adipose tissue and liver, since it was shown that ER stress led to the induction of death signaling pathways. In turn, decreased expression of CHOP and PERK in muscles of EMS horses, as opposed to adipose tissue and liver, might be positively correlated with observed higher expression of IL-13 transcripts in muscles. It was documented that interleukin 4 (IL-4)/ interleukin 13 (IL-13) signaling was suppressed during ER stress by eIF2α-dependent regulation of the C/EBPβ transcription factor, while IL-13 played an unexpected role in regulating glucose homeostasis by gluconeogenesis modulation [31]. This in turn may suggest that muscles of EMS horses, by reduction of ER stress and simultaneous induction of IL-13 expression, are protected against p53-induced cell death, as opposed to adipose tissue or liver. Moreover, a decreased ratio of Bcl-2/BAX indicated increased apoptosis in muscle and adipose tissue in EMS horses. Interestingly, no significant differences were noted in the liver samples. This may indicate that liver undergoes apoptosis via the extrinsic, not the intrinsic, pathway. This correlates with observations claiming that, due to increased expression of death receptor in hepatocytes, apoptosis in the liver is triggered by the extrinsic pathway [32]. However, that thesis needs to be supported by further research.

Insulin resistance, obesity and diabetes are known to be potent factors inducing autophagy, a mechanism that rescues stressed cells from death. Here, we found induced autophagy in the liver, however not in adipose tissue and muscles of EMS horses. We observed significantly higher levels of Beclin-1, LC-3, PI3K and mTOR transcripts in the liver of EMS horses when compared to adipose tissue and muscles. The observed upregulation of these transcripts, which are master components of autophagy, clearly indicated the induction of autophagy. Furthermore, transmission electron microscopy (TEM) allowed visualizing the formation of autophagic double membrane vacuoles that accumulate in hepatocytes around lipid droplets. Interestingly, a significant reduction of LAMP2 expression in muscles, adipose tissue and liver was observed in EMS horses. Lysosomes are important organelles involved in cell death, exocytosis, endocytosis/phagocytosis, as well as autophagy. Their activity and/or dysfunction have been reported in the course of diabetes, however, in the brain of rodents [33]. Lysosomal dysfunction can be caused by lipid accumulation in muscles, adipose tissue and liver of EMS horses, which finally impairs the degradation of carbohydrates, proteins, nucleic acids or cellular debris. Thus, the increased glucolipotoxicity leads to ER stress via oxidative stress, which in turn impairs mitochondrial biogenesis and dynamics. We observed significant upregulation of PARKIN in the liver and adipose tissue of EMS horses, which strongly correlated with progressive ER stress (CHOP and PERK upregulation) observed in these tissues. PARKIN is an E3 ubiquitin-protein ligase that together with PTEN-induced putative kinase 1 (PINK1) is involved in mitochondrial control in the course of mitophagy, thereby promoting cell survival. The enhanced expression of PARKIN in adipose tissue and liver in EMS horses strongly correlates with the observed p53 upregulation, which indicates the apoptotic nature of these adipocytes and hepatocytes. Furthermore, the TEM investigation revealed, typical for mitophagy, impaired mitochondrial cristae and mitophagosome formation. Interestingly, although we did not find enhanced PARKIN expression and mitophagosome formation in muscle samples of EMS horses, mitochondria showed severe ultrastructural changes. It can be speculated that another mechanism may exist in muscles that clears the mitochondrial debris form cellular cytosol. Moreover, we have found enhanced expression of FIS in both the liver and adipose tissue, but not in muscles of EMS horses. This observation positively correlates with lower expression of p21 and p53 in muscles, which may suggest that muscles are least apoptotic under EMS condition when compared to other tested tissue, i.e., adipose tissue and liver. Mitochondrial fission has been described as a mechanism that regulates both mitochondrial bioenergetics and function through changes in proton leakage and membrane potential [34]. It was reported that the inhibition of mitochondrial fission led to a reduction of diabetes-induced oxidative stress and can therefore be a potent therapeutic target [35,36].

In this study, we have shown for the first time that adipose tissue and liver are affected by progressive p53-related apoptosis in EMS and that together with ER stress they induce autophagy to protect the cells against glucolipotoxicity. Moreover, the mitophagy described in these tissues is probably a repair mechanism for maintaining cellular energy and survival. In contrast to these findings, we have found that muscles in EMS condition are least exposed to p53-related apoptosis, and another mechanism than autophagy/mitophagy must exist to protect insulin-resistant cells against apoptosis. Further detailed research is strongly required to explore this issue, since it may be useful in developing future clinical therapy of EMS.

## 4. Materials and Methods

All reagents used in this experiment were purchased from Sigma-Aldrich (Poznan, Poland), unless indicated otherwise.

### 4.1. Horse Selection and EMS Diagnosis

Eight EMS and eight healthy horses were selected from a slaughterhouse located in Rawicz, Poland, and samples of liver, muscles and adipose tissue were collected post mortem. The horses, which were qualified for that research, were Polish warm-blood horses of both sex, aged between 9 and 14 years old. The EMS was diagnosed on the basis of criteria established in the 2010 American College of Veterinary. EMS horses were characterized on the basis of insulin dysregulation (ID), body condition scoring (BCS)regional adiposity, weigth (Wt) and history of laminitis. The body weight was measured on an electronic agriculture platform (BOSCH, Eisenach, Germany). The BSC were evaluated and determined by three independent trained persons according to the Henneke scoring system, where 1 represents an extremely emaciated animal and 9 represents an extremely obese animal. The crest neck was also evaluated by the same investigators on the basis of the cresty neck score (CNS) system where “0” represents reduced neck crest and “5” represents a crest so large it permanently droops to one side of the neck. In all qualified horses, the oral sugar test (OST) was performed as described in [37]. After oral sugar (Karo) administration at a dose of 0.15 mL/kg, blood samples were collected for glucose and insulin measurement. The glucose level were detected at the following time points: “0” and “60” and “120” minutes after Karo administration. A fasting insulin level that was >100 milliinternational units per milliliter (mIU/mL) was considered as hyperinsulinemia, and increased insulin (>150 mU/mL) 60 min post-administration of Karo was considered diagnostic of ID. All samples were collected between 4 and 9 a.m. In Table 1 are presented the data from OST, baseline serum insulin level, fasting insulin and insulin level after 60 min post-oral sugar administration. The analysis of blood glucose was performed by means of a glucometer (Warsaw, Poland) and the insulin concentration was measured using a commercially available kit Mercodia (Equine Insulin ELISA, Uppsala, Sweden, No. 10-1205-01) validated by the Department of Experimental Biology (Wroclaw Environmental and Life Science, Wroclaw, Poland) used on equine serum samples according to the manufactures’ procedure. The measurements were repeated in 3 duplicates by the same technicians. 

### 4.2. Collection of Muscle, Adipose and Liver

Post mortem muscle, subcutaneous adipose tissue and liver samples were collected from healthy horses and horses suffering from equine metabolic syndrome. Immediately after collection, tissues were placed in sterile Hanks’s Balanced Salt Solution (HBSS) and transported to Wroclaw University, Department of Experimental Biology.

### 4.3. Isolation of Proteins from Muscle, Adipose and Liver

Muscle, adipose and liver tissue samples were homogenized and then incubated in RIPA buffer containing 0.001% of protease inhibitor cocktail for 1 h on ice. After incubation, samples were centrifuged for 20 min at 10,000 rpm at 4 °C. The supernatant was collected and stored at −20 °C. The protein concentration was measured by the Pierce™ BCA Protein Assay Kit (Life Technologies, Warsaw, Poland).

### 4.4. Western Blotting

The presence of specific protein in samples was determined by Western blotting. Thirty micrograms of protein were used for each sample. SDS-PAGE was performed at 100 V for 90 min in Tris/glycine/SDS buffer. Proteins were transferred onto a polyvinylidene difluoride membrane (PVDF) (Bio-Rad, Hercules, CA, USA) using a transfer apparatus at 100 V for 1 h at 4 °C in Tris/glycine buffer. After transfer, the membrane was washed with Tris/NaCl/Tween buffer (TBST) and blocked overnight at 4 °C with 5% nonfat milk in TBST. Afterwards, the membrane was washed with TBST and incubated with primary antibody for 2 h: βAKT (Sigma-Aldrich, A5441), PARKIN (Novus, Littleton, CO, USA, NB100-91921) and caspase-3 (Invitrogen, Carlsbad, CA, USA, 437800) at a dilution of 1:500. Then, the membrane was washed with TBST, and the solution of secondary antibody was added. After 2 h incubation, the membrane was washed with TBST and incubated with BCIP^®^/NBT-Purple Liquid Substrate for 15 min. The reaction was stopped by washing the membrane with water. 

### 4.5. ELISA

The total concentration of proteins in tissue homogenates was determined with enzyme-linked immunosorbent assay (ELISA) for IL-1β (My Biosource, San Diego, CA, USA), IL-10 (My Biosource, San Diego, CA, USA) and TNF-α (Thermo Fisher, Warsaw, Poland). Assays were performed in accordance with the manufacturer’s protocol. Each sample was prepared in duplicate. Spectrophotometric determination was performed with Epoch BioTek^®^ (Winooski, VT, USA).

### 4.6. Immunofluorescence and TEM

For immunofluorescence staining, samples were fixed in 4% paraformaldehyde and cryo-protected in sucrose. Frozen sections (10 µm) were cut on a cryostat and thaw mounted on glass slides. Fixed sections were incubated overnight in buffer containing: 0.5% saponin, 0.2% Triton X100, 0.1% sodium azide, 2% goat serum and antibody (1:200, PARKIN, Novus, Littleton, CO, USA, NB100-91921 and LAMP2, Abcam, Cambridge, UK, ab25631). The next day, specimens were washed with PBS, three times for 10 min, followed by a 2-h incubation with Alexa Fluor 488-labelled antibodies. Cell nuclei were counterstained with DAPI (4′,6-diamidino-2-phenylindole). Samples were next examined under a confocal microscope (Cell Observer, Zeiss, Oberkochen, Germany).

To perform transmission electron microscopy analysis, samples were prepared as described previously [38]. They were collected and fixed with 2.5% glutaraldehyde. Then, they were washed three times with distilled water and incubated for 2 h with 1% osmium tetroxide and counterstained with lead citrate and uranyl acetate, dehydrated in a graded series of acetone and embedded using an Agar Low Viscosity Resin Kit (Agar Scientific Ltd., Essex, UK). The observations were carried out using FE-STEM Auriga60 at 20-kV filament tension.

### 4.7. Oxidative Stress Factors in Serum Analysis

Serum was collected from horses and subjected to further analysis. Oxidative stress factors were measured as described previously [39]. Briefly, nitric oxide concentration was evaluated with the Griess Reagent Kit (Life Technologies, Warsaw, Poland) and extracellular superoxide dismutase (Cu-ZnSOD-3) by the SOD Assay Kit in accordance with the manufacturers’ instructions. The level of reactive oxygen species (ROS) was estimated with a 2’,7’-dichlorodihydrofluorescein diacetate (H2DCF-DA, Life Technologies, Warsaw, Poland) solution following the manufacturers’ protocol. Spectrophotometric measurements were carried out using Epoch BioTek^®^ (Winooski, VT, USA). 

### 4.8. Analysis of Genes Expression

Muscles, adipose and liver tissue samples were washed with HBSS and cut into small pieces by means of surgical scissors and a scalpel. Samples were homogenized with TRI reagent. The total RNA was isolated using the phenol-chloroform method described previously by Chomczynski and Sacchi, 1987. The concentration and purity of isolated RNA samples was measured by absorbance measurement with a spectrophotometer (Epoch BioTek^®^, Winooski, VT, USA) at a 260-nm wave length. Preparation of DNA-free RNA and transcription gDNA-free total RNA to a complementary DNA (cDNA) was performed with RevertAid RT Kit (Thermo Scientific, Warsaw, Poland). All of the procedures were performed according to the manufacturers’ instructions. Quantitative real-time polymerase chain reaction (qRT-PCR) was performed using 5 μL of cDNA in a total volume of 20 μL by means of SensiFast SYBR (Bioline, Cincinnati, OH, USA). The reaction was performed at a 2.5 µM final concentration of primers. Primer sequences are presented in Table 1. qRT-PCR was performed by means of the CFX Connect™ Real-Time PCR Detection System (Bio-Rad, Hercules, CA, USA) in the following conditions: 95 °C for 2 min, followed by 50 cycles in 95 °C for 15 s, 60 °C for 15 s and 72 °C for 5 s. After every cycle, single fluorescence measurement of products was performed. Sequences of primers are listed in Table 2. The mRNA levels were normalized relative to the glyceraldehyde 3-phosphate dehydrogenase (GAPDH) as a housekeeping gene. The data were analyzed by the 2^−ΔΔ*C*t^ method. Moreover, we evaluated the ratio between Bcl-2 and BAX expression in each group by dividing the relative expression of Bcl-2 by the relative expression of BAX. 

### 4.9. Statistics

Results are shown as box plots of at least three independent experiments, measured as triplicates or more. Statistical significance was determined using the un-paired *t*-test (Prism5.04; GraphPad Software, La Jolla, CA, USA). *p* < 0.05 was considered statistically significant.

## 5. Conclusions

In presented research, we have shown for the first time that adipose tissue and liver are affected by progressive p53-related apoptosis in EMS. Due to combination of autophagy and ER stress, triggering of autophagy and mitophagy in those tissues was observed as a protective mechanism against glucolipotoxicity. Activation of those processes may serve as repair mechanism for maintaining cellular energy and survival. On the other hand, muscles in EMS condition are least exposed to apoptosis, and distinct mechanism than autophagy/mitophagy must exist to protect insulin-resistant cells against apoptosis.

## Figures and Tables

**Table 1:** Characterization of equine metabolic syndrome (EMS) horses and healthy (control) horses.

Group	Age	Baseline Serum Insulin; (mIU/mL)	Insulin (mIU/mL) 60 min Post Oral Sugar Administration	Baseline Glucose (mg/dL)	Glucose 60 min Post Oral Sugar Administration (mg/dL)	BCS	CNS	LEP (ng/mL)	Wt (Kg)
EMS	12 ± 2	180 ± 20	260 ± 11	72 ± 11	163 ± 26	7.2 ± 0.2	3.9 ± 0.7	4.89 ± 1.2	680 ± 30
Control	11 ± 2	13 ± 4	36 ± 3	84	88	6.4±0.3	1.9±0.6	1.1±0.6	590 ± 20

**Table 2:** Sequences of primers used in RT-PCR.

No.	Gene Name	Primer Sequences		Amplicon Size	Accession Number
1	*AKT*	Forward 5′ → 3′	AAGGAGATCATGCAGCACCG	180	XM_014854427.1
		Reverse 5′ → 3′	CTCCATCGTGTCGTCTTGGT		
2	*BAX*	Forward 5′ → 3′	GCCAGCAAATTGGTGCTCAA	260	XM_014830923.1
		Reverse 5′ → 3′	AGCAGTCACTTCCATGGCTC		
3	*Bcl-2*	Forward 5′ → 3′	TTCTTTGAGTTCGGTGGGGT	164	XM_014843802.1
		Reverse 5′ → 3′	GGGCCGTACAGTTCCACAA		
4	*Beclin-3*	Forward 5′ → 3′	GATGCGTTATGCCCAGATGC	233	XM_014833759.1
		Reverse 5′ → 3′	AACGGCAGCTCCTCTGAAAT		
5	*CHOP*	Forward 5′ → 3′	AGCCAAAATCAGAGCCGGAA	272	XM_014844003.1
		Reverse 5′ → 3′	GGGGTCAAGAGTGGTGAAGG		
6	*FIS*	Forward 5′ → 3′	GGTGCGAAGCAAGTACAACG	118	XM_014854003.1
		Reverse 5′ → 3′	GTTGCCCACAGCCAGATAGA		
7	*GLUT-4*	Forward 5′ → 3′	AAGCCCTCGCTACCTCTACA	210	NM_001081866.2
		Reverse 5′ → 3′	TGCAGCACAACTGCAATGAC		
8	*IL-1β*	Forward 5′ → 3′	TATGTGTGTGATGCAGCTGTGC	352	XM_014852743.1
		Reverse 5′ → 3′	GGCCACAGGTATCTTGTCAGT		
9	*IL-4*	Forward 5′ → 3′	TGACTGTAGCGGATGCCTTT	129	XM_014856772.1
		Reverse 5′ → 3′	GTCCGCTCAGGCATTCTTTG		
10	*IL-10*	Forward 5′ → 3′	TGTTGTTGAACGGGTCCCTG	242	NM_001082490.1
		Reverse 5′ → 3′	ACTCTTCACCTGCTCCACTG		
11	*IL-13*	Forward 5′ → 3′	AGCTGGTCAACATCACCCAG	150	XM_014730431.1
		Reverse 5′ → 3′	GCATCTTCCGCGTGTTTTGG		
12	*IR*	Forward 5′ → 3′	CCGTTTGAGTCTGAGGGGTC	254	XM_014862015.1
		Reverse 5′ → 3′	ACCGTCACATTCCCGACATC		
13	*IRS*	Forward 5′ → 3′	CTGCTGGGGGTTTGGAGAAT	254	XM_014862015.1
		Reverse 5′ → 3′	TAAATCCTCACTGGAGCGGC		
14	*LAMP2*	Forward 5′ → 3′	GCACCCCTGGGAAGTTCTTA	147	XM_014831347.1
		Reverse 5′ → 3′	ATCCAGCGAACACTCTTGGG		
15	*LC3*	Forward 5′ → 3′	TTACTGCTTTGCTCTGCCAC	213	XM_014835085.1
		Reverse 5′ → 3′	AGCTGCTTCTCCCCCTTGTA		
16	*mTOR*	Forward 5′ → 3′	GGGCAGCATTAGAGACGGTG	221	XM_005607537.2
		Reverse 5′ → 3′	ATGGTTGATTCGGTGTCGCA		
17	*MNF*	Forward 5′ → 3′	AAGTGGCATTTTTCGGCAGG	217	XM_014838357.1
		Reverse 5′ → 3′	TCCATATGAAGGGCATGGGC		
18	*PARKIN*	Forward 5′ → 3′	TCCCAGTGGAGGTCGATTCT	218	XM_014858374.1
		Reverse 5′ → 3′	CCCTCCAGGTGTGTTCGTTT		
19	*PERK*	Forward 5′ → 3′	GTGACTGCAATGGACCAGGA	283	XM_014852775.1
		Reverse 5′ → 3′	TCACGTGCTCACGAGGATATT		
20	*PINK1*	Forward 5′ → 3′	GCACAATGAGCCAGGAGCTA	298	XM_014737247.1
		Reverse 5′ → 3′	GGGGTATTCACGCGAAGGTA		
21	*PI3K*	Forward 5′ → 3′	GACTTGCACTTGGGTGACATA	152	XM_014855332.1
		Reverse 5′ → 3′	TAAGTTCCCGGAAAGTCCCC		
22	*P21*	Forward 5′ → 3′	GAAGAGAAACCCCCAGCTCC	241	XM_014853747.1
		Reverse 5′ → 3′	TGACTGCATCAAACCCCACA		
23	*P53*	Forward 5′ → 3′	TACTCCCCTGCCCTCAACAA	252	U37120.1
		Reverse 5′ → 3′	AGGAATCAGGGCCTTGAGGA		
24	*RICTOR*	Forward 5′ → 3′	CTCCACATCGCGAGTCTGTC	166	XM_014738100.1
		Reverse 5′ → 3′	ATCCAATTCAGCTCGCCCAA		
25	*RBP4*	Forward 5′ → 3′	AAGGGTCCAATCTGGCACG	172	NM_001081951.1
		Reverse 5′ → 3′	CAAGTCCTGGCCTAGTCAGC		
26	*SREBP1C*	Forward 5′ → 3′	TCAGCGAGGCGGCTTTGGACAG	80	XM_008542859.1
		Reverse 5′ → 3′	CATGTCTTCGATGTCGGTCAG		
27	*S6K1*	Forward 5′ → 3′	GAGACAGGGAAGCTGAGGACAT	245	XM_014856960.1
		Reverse 5′ → 3′	ACCAAGTACCCGAAGTAGCTC		
28	*GAPDH*	Forward 5′ → 3′	GATGCCCCAATGTTTGTGA	250	XM_014866500.1
		Reverse 5′ → 3′	AAGCAGGGATGATGTTCTGG		

AKT—RAC-α serine/threonine-protein kinase; BAX—Bcl-2-associated X protein; Bcl-2—B-cell lymphoma 2; Beclin-3; CHOP—C/EBP homologous protein; FIS—mitochondrial fission 1 protein; GLUT-4—glucose transporter 4; IL-1β—interleukin 1 β; IL-4—interleukin 4; IL-10—interleukin 10; IL-13—interleukin 13; IR—insulin receptor; IRS—insulin receptor substrate; LAMP2—Lysosome-associated membrane protein 2; LC-3—microtubule-associated protein 1A/1B-light chain 3; mTOR—the mechanistic target of rapamycin; MNF—mitofusin 1; PARKIN—parkin ligase; PERK—eukaryotic translation initiation factor 2-α kinase 3; PINK1—PTEN-induced putative kinase 1; PI3K—phosphatidylinositol-4,5-bisphosphate 3-kinase; p21—cyclin-dependent kinase inhibitor 1; p53—tumor protein p53; RICTOR—rapamycin-insensitive companion of mammalian target of rapamycin; RBP4—retinol binding protein 4; SREBP1C—sterol regulatory element binding protein 1c; S6K1—Ribosomal protein S6 kinaseβ-1; GAPDH—glyceraldehyde 3-phosphate dehydrogenase.

## Abbreviations

BCS	Body condition score
CNS	Cresty neck score
EMS	Equine metabolic syndrome
Wt	Weight
AKT	RAC alpha serine/threonine-protein kinase
BAX	Bcl-2-associated X protein
Bcl-2	B-cell lymphoma 2
CHOP	CCAAT-enhancer-binding protein homologous protein
EMS	Equine metabolic syndrome
ER	Endoplasmic reticulum
FIS	Mitochondrial fission 1 protein
GAPDH	Glyceraldehyde 3-phosphate dehydrogenase
GLUT-4	Glucose transporter 4
IL-1β	Interleukin 1β
IL-10	Interleukin 10
IL-13	Interleukin 13
IL-4	Interleukin 4
IR	Insulin receptor
IRS	Insulin receptor substrate
LAMP2	Lysosome-associated membrane protein 2
LC-3	Microtubule-associated protein 1A/1B-light chain 3
MCP-1	Monocyte chemotactic protein 1
MNF	Mitofusin 1
mTOR	The mechanistic target of rapamycin
NO	Nitric oxide
OS	Oxidative stress
p21	Cyclin-dependent kinase inhibitor 1
p53	Tumor protein p53
PARKIN	Parkin ligase
PERK	Eukaryotic translation initiation factor 2-alpha kinase 3
PI3K	Phosphatidylinositol-4,5-bisphosphate 3-kinase
PINK1	PTEN-induced putative kinase 1
RBP4	Retinol binding protein 4
RICTOR	Rapamycin-insensitive companion of mammalian target of rapamycin
ROS	Reactive oxygen species
S6K1	Ribosomal protein S6 kinase beta-1
SOD	Superoxide dismutase
SREBP1C	Sterol regulatory element binding protein 1c
TNF-α	Tumor necrosis factor α
